# Parents' and carers' views about emollients for childhood eczema: qualitative interview study

**DOI:** 10.1136/bmjopen-2016-011887

**Published:** 2016-08-19

**Authors:** M Santer, I Muller, L Yardley, S Lewis-Jones, S Ersser, P Little

**Affiliations:** 1 Primary Care & Population Sciences, Aldermoor Health Centre, University of Southampton, Southampton, UK; 2 Ninewells Hospital & University of Dundee, Dundee, UK; 3 University of Leeds, Leeds, UK

**Keywords:** QUALITATIVE RESEARCH, PRIMARY CARE

## Abstract

**Objective:**

Leave-on emollients form the mainstay of eczema treatment, but adherence is poor. We aimed to explore parents’/carers' views on effectiveness and acceptability of leave-on emollients for childhood eczema through secondary analysis of data from 2 qualitative data sets.

**Setting:**

Study 1 recruited through mail-out from 6 general practices in southern England. Study 2 recruited from a feasibility trial of an intervention to support eczema self-care in 31 practices in the same area.

**Participants:**

Study 1 included 28 interviews with carers of children aged ≤5 years with eczema. Study 2 included 26 interviews with carers of children aged ≤5 years with eczema.

**Methods:**

Interviews followed semistructured guides: study 1 explored carers' understandings around eczema treatments in order to develop a web-based self-care support intervention; study 2 explored carers' understandings of eczema and eczema treatments after using the intervention. Interviews were carried out face to face or by telephone, audio-recorded and transcribed. Secondary analysis of data from both studies focused on views and experiences of emollient use. Data were analysed using an inductive thematic approach facilitated by NVivo V.10 software.

**Results:**

In study 1, most participants felt emollients improved eczema but held mixed views about long-term use to prevent flare-ups. In study 2, where carers had used the web-based intervention, all participants held positive views about long-term emollient use. In both studies, participants expressed a range of preferences about emollient ‘thickness’; some felt that ‘thick’ emollients (ointments) were most effective, while others found these difficult to use. Carers described a process of ‘trial and error’, trying emollients suggested by professionals, friends and family, or bought over-the-counter. Carers expressed a need for understanding differences between products and their effective use.

**Conclusions:**

Providing a rationale for long-term emollient use and choice of emollients could help improve adherence and help families gain more rapid control of eczema.

**Strengths and limitations of this study**

- This is one of the first qualitative studies to explore parents'/carers' views, experiences and understandings of the use of leave-on emollients for childhood eczema.
- A strength of this study is that it draws on two study groups with different experiences of eczema education and support, giving a diversity of views and understandings.
- A limitation is that this is a secondary analysis: if exploration of emollient use had been our primary aim, we may have questioned participants about this in still greater depth.

## Background

Childhood atopic eczema, or atopic dermatitis, is very common, affecting over 20% of children aged ≤5 years at some point.^1^ Childhood eczema has significant impact on quality of life for children and their families, particularly due to sleep disturbance and itch.^2^ ^3^ However, non-adherence to long-term treatments for eczema is thought to be the main barrier to effective treatment^4^ ^5^ and carers express frustrations with the advice they receive.^6–8^

Guidelines suggest that emollients (non-cosmetic moisturisers) form the mainstay of treatment for eczema and should be used regularly by all adults and children with eczema, in addition to other treatments (such as topical corticosteroids) for acute flare-ups and when in remission to prevent flare-ups.^4^ ^5^

This article relates to leave-on, or directly applied, emollients (as opposed to soap substitute or bath emollients). Leave-on emollients are thought to provide a protective layer over the skin, decrease moisture loss and occlude against irritants and are widely prescribed at a cost of over £71 million per year to the National Health Service in England.^9^ Consensus guidelines suggest that ointments (thicker formulations such as white soft paraffin) can be used when the skin is very dry and creams and lotions when the skin is less dry.^10^ However, there is little evidence regarding the relative effectiveness of different emollients; guidelines conclude that prescribing should be based on informed patient choice,^11^ or that, ‘The correct emollient is the one that the child will use’.^4^ ^5^

Qualitative studies have found that patients dislike conventional treatments for eczema^12^ and are concerned about a lack of information about treatments,^6^ although existing research focuses mainly on topical corticosteroids.^13^ One qualitative study has included brief discussion of emollient use, reporting that some participants felt emollients provoked rather than prevented eczema and some expressed disappointment at the limits of professional knowledge and evidence base for the eczema management, for instance stating that, ‘This “trial and error” is a very primitive form of medicine’.^8^

In the quantitative literature, there is also more information on topical corticosteroid use than emollient use,^14–18^ although one study found that most carers of children with eczema did not believe that emollients prevented flare-ups, even after receiving instruction about this in secondary care.^19^

A recent feasibility trial^20^ and subsequent plans for a full-scale trial have raised questions about carers' views of emollient use for childhood eczema. In light of the paucity of data on this topic, we present unreported findings from a secondary analysis of data collected as part of two qualitative studies carried out among carers of children with eczema. Study 1 was a qualitative interview study with the primary aim of exploring views/experiences around treatment for childhood eczema, in order to inform the development of a web-based intervention to support self-care. Study 2 was a qualitative study among carers who had used the web-based intervention, as part of its evaluation. We previously reported findings from study 1 showing that some carers did not believe emollients to be effective and that a minority viewed emollients as ‘unnatural’ and potentially harmful if used long term.^7^ We also previously reported that carers described mixed feelings about the process of ‘trial and error’,^7^ a process that is often necessary to find an emollient that suits the child. These are complex topics where carers held a wide range of views and understandings. Here, we explore these in greater depth and more fully describe and explore carers' views about the effectiveness, acceptability and choice of leave-on emollients.

## Methods

The primary aim of study 1 was to explore carers' views and experiences of eczema treatments, in order to develop a web-based intervention.^7^ ^21^ ^22^ Study 2 was carried out among carers who had used the web-based intervention, primarily as part of a process valuation and to inform further refinement of the intervention.^23^ Data from both studies were reanalysed as the basis for this article using an inductive thematic approach facilitated by NVivo V.10 software.

### Study 1

#### Population

Staff from six general practices in the south of England searched their databases for children aged ≤5 years with a recorded diagnosis of eczema and posted invitations to ‘parent or carer of [name of child]’. Carers were asked to return a reply slip to the research team and, if they were happy to participate and eczema was still a problem, they were telephoned to arrange an interview.

#### Data collection

We purposively sampled participants with a range of parental and child ages and differing geographical areas. Informed consent was sought prior to the interviews, which were semistructured and followed an interview guide (box 1). Interviews lasted 30–60 min, taking place in participants' homes except for one where the participant chose to be interviewed at her health centre. Interviews were carried out by MS, CH and HB from December 2010 to May 2011. MS and HB are general practitioners (GPs), but no participants were registered at practices where they worked. CH was a medical student. All introduced themselves as ‘researchers’. In a few cases where participants asked after the researcher's professional background or where participants had significant information needs, these were discussed after the end of the interview. Interviews were audio-recorded, transcribed and transcripts were checked against recordings.

Box 1 **Study 1 Interview guide**

▸ Can you tell me a bit about your child's eczema to start with?

- How does it affect your child?
- How does it affect family life?
- Can you tell me about what it was like when it first started?
- Can you tell me about how it has been more recently?

▸ Can you tell me about any professional advice you have had about it?

▸ Can you tell me about any other kinds of advice or information you have had? (other people? books, leaflets, internet?)

▸ What kinds of things do you do to try to cope with the eczema?

- How do you feel about that?
- Can you tell me about any problems you have had with that?

▸ Do you do anything else to try and help your child's eczema?

- Can you tell me about any difficulties you have doing these things?
- If so, how do you get round these difficulties?

▸ How do other members of the family feel about the eczema?

▸ What else do you think would be helpful or would have been helpful in the past?

### Study 2

The interviews were carried out in the context of a feasibility trial that randomised participants to three groups: (1) web-based intervention plus usual care, (2) web-based intervention plus healthcare professional support plus usual care and (3) usual care alone. The web-based intervention included information about rationale for emollient use, videos on emollient use and a ‘2-week challenge’ for carers to use emollients regularly and judge the difference for themselves (box 2). Further details are reported elsewhere.^23^

Box 2 **Overview of web-based intervention in study 2**

On their first visit to the website, users were offered two core modules (‘What is eczema?’ and ‘Emollient moisturisers') before reaching a ‘menu’ of 14 modules, including diet and allergy, topical corticosteroids, involving your child in treatment, bath-time, sleep problems and managing scratching. On subsequent visits to the website, users would go straight to the ‘menu’ of modules.

The ‘2-week challenge’ was an optional component of the ‘emollient moisturisers' module. Users were asked to sign up to using emollients regularly for 2 weeks and were offered a ‘tick sheet’ to help them with this (they were able to alter how many times per day on the tick sheet). As part of the ‘2-week challenge’ they were also offered daily SMS/text messages as reminders.

Some modules included short videos (∼4 min each) illustrating techniques such as applying emollients or bathing a child. Links to these videos were contained in the relevant modules, but there were also links to all the videos in a button on the ‘menu’ screen. Similarly, ‘print sheets' (such as tick charts for children, action plan to take to GP consultation, summary of eczema management for relatives, school or nursery) appeared in the relevant modules, but there were also links to all of these in a button on the ‘menu’ screen.

#### Population

We recruited to the feasibility trial via GP mail-out from 31 practices in the south of England using the same methods as study 1, but with different practices. If carers replied and the eczema was still a problem, they were sent a unique login to complete online consent, questionnaire and randomisation and invited to provide contact details if they were willing to participate in a qualitative interview following participating in the feasibility trial.

#### Data collection

We purposively sampled participants from lower socioeconomic class areas as these were less well represented in study 1, while also seeking a range of ages and geographical areas. Semistructured interviews took place at a location of participant's choice: 15 in participants' homes, 1 at the participant's workplace and 10 by phone. Interviews followed an interview guide (box 3) and lasted 20–60 min. Interviews were carried out by MS (GP) and HS (Medical Student) from November 2012 to February 2013, who introduced themselves as researchers. Interviews were audio-recorded, transcribed and transcripts were checked against recordings.

Box 3 **Study 2 interview guide**

▸ So can I ask, first of all, how did you find taking part in (study of web-based intervention)?

▸ How did you feel about it overall? (How did it go overall?—*only if necessary*)

▸ Has taking part in this changed how you feel about eczema?

- Has it changed how your family feels about the eczema?
- Has it changed anything for your child?

▸ Can you tell me a bit about how you used the website?

- How often did you use it?/Can you remember which sections you looked at?
- Did you do the 2-week challenge? How did that go?
- Was there anything that annoyed you about it, or any information that you were hoping for and didn't find?

▸ What had you been hoping to get from this study before taking part?

NURSE GROUP ONLY

▸ Did you go and see the nurse to discuss the website? If not, why not?

- If yes, how did it go overall?
- What did you discuss at this appointment?
- Did you find this appointment useful?

▸ Did it change how you feel about eczema, or how the eczema is for your child?

NON-NURSE GROUP ONLY

How did you feel about not being offered an appointment with the nurse?

EVERYONE

Is there anything that we haven't discussed that you would like to add?

#### Data analysis

This secondary analysis focuses on the under-researched topic of carers' understandings of emollient use for childhood eczema, particularly issues around effectiveness, acceptability and choice of emollient.

In study 1 and study 2, all data relating to views or experiences of emollients were assigned a broad overall code (by MS and CH in study 1 and MS in study 2: codes discussed with LY and IM). For the secondary analysis informing this article, all data relating to these codes were retrieved for further subcoding (by MS in discussion with IM), although data from the two studies were kept separate in order to retain their different contexts. Data were analysed using inductive thematic analysis.^24^ Subcodes were developed into themes, summaries written for each theme and counterexamples were sought. Data saturation was achieved for the main themes in studies 1 and 2, but the nature of this secondary analysis means that we did not check for saturation of emollients codes during data collection. However, the breadth and depth of data on emollients suggests it is unlikely that further interviews would have yielded substantial new findings. NVivo V.10 software was used to facilitate data handling. Pseudonyms have been assigned in reporting the findings.

## Findings

We present findings on carers' perceptions of whether or not emollients work (for active eczema and to prevent flare-ups) and about which emollients are best, including how carers reach decisions about this. We did not specifically ask interviewees about emollients in either study 1 or study 2, yet all interviewees mentioned emollients when asked about their experiences of caring for childhood eczema.

## Study 1

Invitations were sent to 289 households. We received 70 replies, of which 33 said eczema was no longer a problem, 3 declined for other reasons and 6 were not contactable, resulting in 28 interviews (10% of mail-out) (table 1). All participants in studies 1 and 2 were parents of children with eczema, so we will present our findings with reference to ‘parents’ rather than ‘carers’.

**Table 1:** Study 1 participant characteristics

Participant characteristics	Description		
Interviewee's relationship to child*	27 mothers	4 fathers	
Interviewee's age	Median 36 years (range 26–46 years)		
Age of most affected child	Median 3 years (range 7 months–5 years)		
Severity (parent asked if child's eczema currently mild, moderate or severe)	16 mild	10 moderate	2 severe
Family structure	Number of children in household median 2 (range 1–4); 27 households with both parents, 1 household with mother only		
Interviewees’ employment status*	14 full-time carers; 8 professional; 8 other such as admin or retail; 1 student		
Ethnicity*	25 white British; 3 black or Asian British; 2 white non-British; 1 mixed heritage British		

*Total 31 interviewees from 28 families as 3 couples were interviewed together.

### Do emollients help?

In study 1, we asked participants what treatments they were using for their child's eczema and their views and experiences of using these. All interviewees mentioned emollients. Most interviewees (21 of 28) said that they thought the emollients did help their child's eczema in the short term, but that barriers to regular use meant they did not always use them.

Caroline: If you forget, or we're out, or I haven't got the cream with me or whatever, it's worse the next day. If he's been away to his dad's over the weekend and he's not done it, then I might need to use a bit of hydrocortisone to get it back to that and then we just slap on the Epaderm [ointment] again. It just goes on and on and on.

Some parents described caution about applying emollients to an infant or child, related to a feeling that emollients are ‘unnatural’ or contain ‘chemicals’. Some also expressed a view that the skin becomes ‘used to’ emollients and they lose their effectiveness if used regularly.

Interviewer: You said earlier that you'd been reluctant to use the creams, was that the steroid or all of them?

Hilary: Initially all of them. I had been trying just to keep, you know, just natural products. The prospect of putting on a cream that had 40 chemicals in then, just didn't sit well. But after trying an alternative for a short period of time, I just thought, OK, well, this isn't working and I don't have an option.

Interviewer: OK, and do you think the creams are helping at the moment?

Sam: Sometimes I think they do and sometimes I think they don't. I think where I'm so used to it. Since I've changed to the emulsifier more than when I have done the Epaderm, it works a bit—like I said—his body just gets too used to it.

Some interviewees were less concerned about adverse effects from emollients but were unconvinced about their effectiveness, or about the rationale for continued use of emollients, although most continued to use them.

Sabrina: The steroid certainly works, obviously because they are quite a good one and they are very strong I think, but other than that, I think the others—I'm not sure. I know because it's given by the GP or the dermatologist, so there must be a reason why they give you cream like that but it's—it's really just like a moisturiser, it doesn't make it better, it's just to moisturise the skin, so basically, I'm not sure whether they actually really, really work.

Two of the 31 interviewees felt that the emollient did not work and did not use them. Leyla just felt that they were ineffective, but Mandy felt that leave-on emollients seemed to sting her daughter, so relied instead on bath emollients and regular topical corticosteroids.

Mandy: She has this idea that her creams sting her legs whereas the—I know the steroid cream doesn't do that because it's obviously made to go on broken skin, but we have put moisturiser on her and she's cried and it's been all inflamed.

Although most parents in study 1 said that they thought regular emollients helped their child's eczema, there were more mixed views about the use of emollient when the eczema was in remission. A few parents did indeed feel that emollient helped prevent a flare-up.

Interviewer: And what do you put on her skin at the moment?

Pippa: We just use Unguentum [ointment] constantly, cause I know if I stop using it, it goes really dry again and then it's prone to cracking and flaring up.

More commonly, however, parents in study 1 described using emollients only after symptoms of active eczema, either because they seemed unaware of regular emollient use as a long-term treatment or because they rationalised that this was unnecessary. The following data excerpt is typical:

Tina: I just don't think it helps having constant moisture on your skin… If it doesn't need it, why put it on? Cos I don't put it on the rest of his body, it's only where he gets hot at night, he scratches and then I put it on him.

### Which emollient is best?

Some people were aware of the different ‘thicknesses’ and types of emollient, particularly among parents with longer experience of managing eczema. Interviewees expressed a range of preferences for emollient type; for some, ‘thickness’ seemed to be a barrier to adherence, whereas for others, ‘thickness’ was essential to their view that the emollient was effective and ‘greasiness’ was something that they accepted:

Interviewer: Have there been any problems applying the new cream?

Carrie: No, cause the cream she has is like—Diprobase [cream], it's like it is easier, is not too—cos the other creams, we had a couple, I think it was Epaderm [ointment], I'm not sure, but it was really thick, like Vaseline [ointment], they were quite difficult—they're really thick, greasy, really hard to apply, cos that ruined loads of clothes, so you had to wash things. That was quite hard work at the beginning, I'd say.

Rosie: I found it hard cause I couldn't relieve any of the symptoms. We didn't have the Epaderm [ointment], we had this Doublebase [gel] and it just didn't seem to be working: we were putting loads on her, it just seems to make her greasy, I had to use the hydrocortisone [topical corticosteroid] about, I don't know 3 or 4 times a day sometimes, because, you know her skin was flaring up that much and she seemed to scratch a lot more and sometimes seemed to be in distress about it.

Many expressed confusion about the number of different emollient products available and what the rationale was behind choosing one over another. People were keen to understand more about the different products and sometimes seemed bewildered about why they were prescribed particular emollients.

Interviewer: How many times do you think you've been to [surgery] about it?

Helen: How many creams have I got? [laughter] [long pause] Maybe 4… I think I saw a different person every time. I know I definitely spoke to 2 different doctors and 2 different health visitors… One I was never recommended by that practice, which all my friends seem to get recommended is Diprobase [cream]. And actually whether it probably was illegal but someone did actually give me some to try, but I didn't use it too much cos I kind of thought, oh, it's prescription and maybe they haven't given it to me for a reason. And I even mentioned it to one of the doctors, and they just said, well, I tend to use this so he was never prescribed it. I don't really know why and why not.

### ‘Trial and error’

‘Trial and error’ is a term for the process of trying a number of different emollients until one is found that suits the child. Most interviewees seemed aware of ‘trial and error’ and seemed comfortable with the concept that different preparations might suit different children.

Interviewer: Where did you first get advice from?

Sarah: I think quite a lot of it—trial and error on different creams, so different GPs giving me different things, giving me different advice, going back and asking different things. But the Epaderm is what really seemed to get on top of it and it was just trial and error to see actually what I had to do.

‘Trial and error’ could be initiated by health professionals or by friends, family or parents themselves. In many accounts, ‘trial and error’ had been initiated by the family:

Carrie: I've only found out about different emollients and things like that by investigating it myself; they sort of gave me standard emollients that they prescribe, and it didn't really suit her skin very well… her skin just used to be red after using it. And we changed it to the Balneum which seems to be better, better for her. I've found that on my own.

There were some parents who found the process of ‘trial and error’ from their healthcare provider a negative experience and, as previously reported,^7^ linked this with feeling that this was an insufficient response from health professionals and that they wished for something else.

Interviewer: OK, in what way do you think he could have helped more?

Sam: Well he just gave me more creams, basically, I've had creams since he was born; I've got loads of different creams in the house, I could make a chemist, do you know what you mean.

## Findings from study 2

Of the 143 people who took part in the pilot study, 82 agreed to be contacted for a feedback interview. We approached 45 of these after they had completed follow-up, of whom 14 were uncontactable, 4 declined to be interviewed, 1 gave feedback by email and 26 were interviewed (table 2).

**Table 2:** Study 2 participant characteristics

Interviewee's relationship to child	24 mothers	2 fathers	
Interviewee's age	Median 37 years (range 24–44 years)		
Age of most affected child	Median 3 years (range 14 months–6 years)		
Severity (parent asked if child's eczema currently mild, moderate or severe)	16 mild	7 moderate	3 severe
Family structure	Number of children in household median 2 (range 1–5); 25 households with both parents, 1 household with mother only		
Interviewees’ employment status	5 full-time carers; 8 professional; 13 other such as admin or retail		
Ethnicity	23 white British; 1 black or Asian British; 2 white non-British		

Participants in study 2 were not directly asked about their views of emollients, and yet emollient use arose in response to a question about changes in their child's eczema since using the web-based intervention. Themes relating to emollient use were broadly similar to study 1, in particular reflections on whether emollients worked and which were best, but participants in study 2 held more positive views about regular emollient use to prevent flare-ups and the process of ‘trial and error’.

### Do emollients help?

All participants in study 2 seemed convinced that emollients worked and many spoke about regular use of emollients to prevent flare-ups. Unlike in study 1, no participants spoke about avoiding long-term use. Several parents said that they had not really felt that emollients helped until they used them more frequently and in greater volumes, as a result of using the website. Nicole mentioned that she had taken up the ‘2-week challenge’ section of the web-based intervention (box 2).

Interviewer: And how did it go [2 week challenge]?

Nicole: Brilliant. It just made me use moisturisers a lot more. Before that I had just worked with the eczema when there was a flare up, mostly with the hydrocortisone, but I didn't do anything when the eczema wasn't there. It made me deal with it all the time and that has made an absolute difference, definitely.

Several interviewees commented that their approach to emollient use had altered through using the website. For instance, the website included video demonstrations of how to apply emollient, which were mentioned by some.

Interviewer: Any more information that you'd like to know about it?

David: Okay, so it was—it was about, it was literally about trying different things; the main one was, for me, was sheer quantity, but that was on there, because you saw it. It was like, crikey, you're putting a dirty great handful on, where we were being quite sparing with it.

The website was designed as a behaviour change intervention to increase emollient use and some parents commented that establishing a ‘habit’ of regularly using emollients had led them to observing more benefit from emollients than previously.

Interviewer: So you said that the regular text reminders had made a bit of a difference (P: Yes) with doing the Diprobase, did you see any difference in [youngest child's] eczema with that?

Annie: Yes, yes, it did—it did—it did start to clear, although it certainly—it was really, really super dry. Yes, doing it every day definitely, yes, I should do it every day anyway, if I was a good mother I would. [laughter] But the text [SMS reminder], it was sent at a really good time, it was sent at about six o'clock, so I did their bath and I remembered.

### Which emollient is best?

Interviewees in study 2 said that they particularly valued the section of the intervention that explained about the different types of emollient available, helping them to find one of the right ‘thickness’.

Interviewer: And going back to the website, do you remember which sections you used particularly?

Jo: It was the emollients one, about all the different emollients, because we tried so many of them, that was the main one that I used all the time, to find a cream that he liked, and that we hadn't used before, and I find some of them are quite watery, and we wanted to find one that was quite, actually felt like it was doing something to his skin, you know.

Although there was information about different emollients on the website, some participants in study 2 felt they would have liked even more, again suggesting the importance of this topic to parents.

Interviewer: Is there anything you would have liked more information on?

Petra: Well I suppose I would have liked a list of creams, lotions, actual names of things. I think I did get a bit confused, like I've got Dermol lotion, I've got Dermol cream, well what's the difference? Because you can go to the doctor and they just prescribe something, but you don't really know—is it a cream, is it a lotion, is it a -? And I only know that from the website. In a way it's sort of made me understand about the different products. I should imagine for people who've got children with even worse eczema, it's quite baffling, you know.

An unanticipated effect of the intervention was that it prompted some parents to seek out different emollients. Some had not realised that they could ask for a different emollient, or that they were aware of this but did not feel confident to reconsult just for this issue. Hayley said she had not really appreciated that a different emollient might be more beneficial until she tried the ‘2-week challenge’.

Interviewer: And did you do the two-week challenge at all, do you remember?

Hayley: Yes, yes I did. That sort of highlighted that the cream that he was having definitely wasn't really effective; in fact in some ways it seemed to sort of irritate him more. It came up in more redness when he was using that. So I went back to the doctor and swapped to another cream and I found obviously that then doing the two-week challenge on that cream, we found that one was definitely a lot better for him.

## Discussion

This is the first article we are aware of that focusses on carers' views about the use of leave-on emollients for childhood eczema. We found that most carers of children with eczema said that emollients helped their child's eczema in the short term, although they held mixed views about the use of emollients in the long term to prevent flare-ups. Carers who had followed a self-care support intervention expressed more positive views towards using emollients to prevent flare-ups.

Although these studies were not designed to test the effectiveness of the self-care support intervention, it is interesting that carers who had used it spoke about their views and habits having changed due to information that they had received. This suggests that reluctance to use emollients in the long term may be overcome by appropriate information and behaviour change support (such as videos to model emollient use and prompts to establish emollient use as ‘habit’).

A range of views and preferences were expressed about ‘thickness’ of emollient, with an apparent trade-off between perceived effectiveness of ‘thicker’ preparations against ease of application of ‘thinner’ preparations. There was widespread interest in obtaining more information about different emollient products available and the rationale for choosing one emollient preparation over another.

### Findings in the context of existing research

Our findings echo previous descriptions of substantial unmet information needs among carers of children with eczema, particularly their wish for better information about treatments.^6^ There is extensive literature on carers' concerns about topical corticosteroids^14–16^ ^18^ ^25^ ^26^ and our study highlights that some carers also have concerns around emollients. These findings highlight the need for education or self-care support for carers of children with eczema, although there is currently a scarcity of proven interventions to support this.^27^ ^28^

Our findings highlight the need for choice of emollient, as there were substantial differences in preferences for different ‘thicknesses’, reflecting the mantra that, ‘The correct emollient is the one that the child will use’.^4^ ^5^ This choice is implemented as a ‘trial-and-error’ process of trying different emollients sequentially in primary care, although this has previously been reported as a frustrating process for some.^8^ ^21^

### Strengths and limitations

One strength of this study is that we used a population-based sampling frame (as almost all UK residents are registered with a GP) in order to seek a wide range of views. However, our samples include relative few fathers, ethnic minorities and low income or single parent households and the response rate to participate in interviews was low.

The findings are strengthened by having been drawn from two study groups with different experiences of eczema education and support, highlighting that carers who had received self-care support seemed happier to use emollients in the long term. A limitation is that this is a secondary analysis: if exploration of emollient use had been our primary aim, we may have questioned participants about this in greater depth and more fully explored perceptions of emollients and how these developed.

### Implications for research

Questions remain about how the ‘trial-and-error’ process can be shortened and made less frustrating for carers. It has been suggested that a tray of different emollients should be set up in clinic to enable carers to make a choice about different emollients.^29^ In primary care, where the majority of children with eczema are managed, this may be difficult to achieve in practice, especially when trial-and-error learning in the home setting is needed. Furthermore, there is no evidence at present to show that the process of offering patients or carers a range of emollients is beneficial. Further evidence is needed about how this process can be improved and made less frustrating for carers.

### Implications for practice

Participants who had received more information about eczema and explanation of rationale for emollient prescribing appeared more convinced of the need for long-term emollient use to prevent flare-up. This reiterates the importance of providing carers with clear accessible information about eczema and an explanation of the rationale behind continued use of emollient and choice of emollient in promoting adherence. Interventions that help promote emollient as a ‘habit’ could be particularly effective, as could promotion of concepts such as a ‘2-week challenge’ of regular emollient use, which allows carers to explicitly evaluate the outcomes of the emollient they are using. Providing such support or signposting carers towards more reliable sources of evidence-based online resources is an essential part of eczema management. (See box 4 for current online resources for carers of children with eczema.)

Box 4 **Online resources for carers of children with eczema**

Websites

NHS Choices http://www.nhs.uk/conditions/Eczema-(atopic)

The National Eczema Society http://www.eczema.org

Nottingham Support Group for Carers of Children with Eczema http://www.nottinghameczema.org.uk

http://www.eczemaadvice.co.uk

YouTube videos

https://www.youtube.com/watch?v=kPBN4_oATEo

http://www.bch.nhs.uk/story/information-video-parents-children-eczema

## Conclusions

Carers value having the choice of emollients and hold varying views about which they find most helpful. However, the process of ‘trial and error’ can be frustrating and carers need a scientific rationale for engaging in the process instead of feeling that it is second rate ‘hit and miss’ clinical management. A clear rationale for selection of specific emollients and techniques for explicitly evaluating treatment outcomes could help them feel more confident, involved and empowered.
